# Single drug biomarker prediction for ER− breast cancer outcome from chemotherapy

**DOI:** 10.1530/ERC-17-0495

**Published:** 2018-03-29

**Authors:** Yong-Zi Chen, Youngchul Kim, Hatem H Soliman, GuoGuang Ying, Jae K Lee

**Affiliations:** 1 Department of Biostatistics and BioinformaticsH. Lee Moffitt Cancer Center and Research Institute, Tampa, Florida, USA; 2 Department of Cancer Cell BiologyTianjin Medical University Cancer Institute and Hospital, National Clinical Research Center for Cancer, Tianjin, People’s Republic of China; 3 Department of Women’s Oncology and Experimental TherapeuticsH. Lee Moffitt Cancer Center and Research Institute, Tampa, Florida, USA; 4 Department of Clinical SciencesCollege of Medicine, University of South Florida, Tampa, Florida, USA

**Keywords:** biomarker, estrogen receptor, chemotherapy, gene expression

## Abstract

ER-negative breast cancer includes most aggressive subtypes of breast cancer such as triple negative (TN) breast cancer. Excluded from hormonal and targeted therapies effectively used for other subtypes of breast cancer, standard chemotherapy is one of the primary treatment options for these patients. However, as ER− patients have shown highly heterogeneous responses to different chemotherapies, it has been difficult to select most beneficial chemotherapy treatments for them. In this study, we have simultaneously developed single drug biomarker models for four standard chemotherapy agents: paclitaxel (T), 5-fluorouracil (F), doxorubicin (A) and cyclophosphamide (C) to predict responses and survival of ER− breast cancer patients treated with combination chemotherapies. We then flexibly combined these individual drug biomarkers for predicting patient outcomes of two independent cohorts of ER− breast cancer patients who were treated with different drug combinations of neoadjuvant chemotherapy. These individual and combined drug biomarker models significantly predicted chemotherapy response for 197 ER− patients in the Hatzis cohort (AUC = 0.637, *P* = 0.002) and 69 ER− patients in the Hess cohort (AUC = 0.635, *P* = 0.056). The prediction was also significant for the TN subgroup of both cohorts (AUC = 0.60, 0.72, *P* = 0.043, 0.009). In survival analysis, our predicted responder patients showed significantly improved survival with a >17 months longer median PFS than the predicted non-responder patients for both ER− and TN subgroups (log-rank test *P*-value = 0.018 and 0.044). This flexible prediction capability based on single drug biomarkers may allow us to even select new drug combinations most beneficial to individual patients with ER− breast cancer.

## Introduction

Breast cancer results in half of a million deaths annually and greatly impacts the quality of life of many millions of patients and their families worldwide (Golubnitschaja *et al*. 2016). It is well known that breast cancer is a heterogeneous disease with a wide range of tumor interheterogeneity and clonal intraheterogeneity (Russnes *et al*. 2011). Currently, breast cancer has been classified into several molecular subgroups such as luminal A & B, Her2 and Triple-negative/basal-like (TN) subtypes (Perou *et al*. 2000). In particular, patients in the ER-negative group, which includes TN and Her2 subtypes, have shown signficantly worse survival outcomes than the other subtypes of breast cancer patients (Prat *et al*. 2015). As ER− breast cancer patients are excluded from hormonal and targeted therapies that are effectively used for other subtypes of breast cancer, neoadjvant chemotherapy is widely used in the management of ER− breast cancer in order to decrease tumor size, eradicate nodal disease or allow surgeons to limit the extent of surgery required (Mittendorf *et al*. 2016). Many recent studies have attempted to predict these patients’ responses to neoadjuvant chemotherapy. Hess *et al.* used a 30-gene pharmacogenomic predictor to predict pathological complete response (pCR) to preoperative weekly paclitaxel and fluorouracil-doxorubicin-cyclophosphimide (T/FAC) chemotherapy (Hess *et al*. 2006). Tabchy *et al*. then evaluated this 30-gene predictor in a multicenter randomized trial, but this model could not successfully predict the chemotherapy response for the patients in the study (Tabchy *et al*. 2010). Horak *et al*. have used single and multigene expression models to predict pCR between doxorubicin-cyclophosphimide (AC) + ixabepilone vs AC + paclitaxel treatments (Horak *et al*. 2013). Iwamoto *et al*. identified multiple gene sets that were significantly associated with pathological complete response on FAC/FEC treatment cohorts (Iwamoto *et al*. 2011). Hatzis *et al*. have developed a 33-gene predictor for ER-positive breast cancer and 27-gene for ER-negative breast cancer, which showed a significant difference of PFS for the patients predicted to response to anthrcyline-based chemotherapy (Hatzis *et al*. 2011).

While these studies have shown potential of genomic biomarkers for predicting outcomes of breast cancer patients treated with chemotherapy, their prediction capabilities were limited to each specific combination chemotherapy, which was used to treat the patients in each study. It is uncertain if these biomarker models developed for specific drug combinations would also be predictive for slightly different combinations or individual drugs included in the original combinations. It is also critical to find most beneficial drugs early for patients with aggressive and metastatic breast cancer as single drugs are often administered for these patients. Ultimately, it will be highly desirable to find optimal combinations of drugs for the heteregenous patient population of ER− breast cancer. In order to examine such a possibility, we have simultaneously developed four single drug biomarker models for paclitaxel, 5-fluorouracil, doxorubicin and cyclophosphamide, which are commonly used in standard chemotherapies for breast cancer. In particular, these biomarker models were developed and obtained based on a common platform of genomic expression data for their practical applications. Our initial single drug biomarkers were discovered based on the microarray expression data on cancer cell lines, which were treated with each drug (Shen *et al*. 2012). In order to link *in vitro* cell line chemosensitivity to breast cancer patients’ chemotherapy response, we then identified and used concordantly expressed biomarkers between the cell lines and *in vivo* patient tumors (Lee *et al*. 2007). We have evaluated and selected our optimal drug models with the patient cohorts enriched with ER− breast cancer. Finally, we have validated these single and combined drug models with two completely independent patient cohorts of ER− breast cancer who were treated with different combination chemotherapies.

## Materials and methods

### Patient data

**Figure 1:**
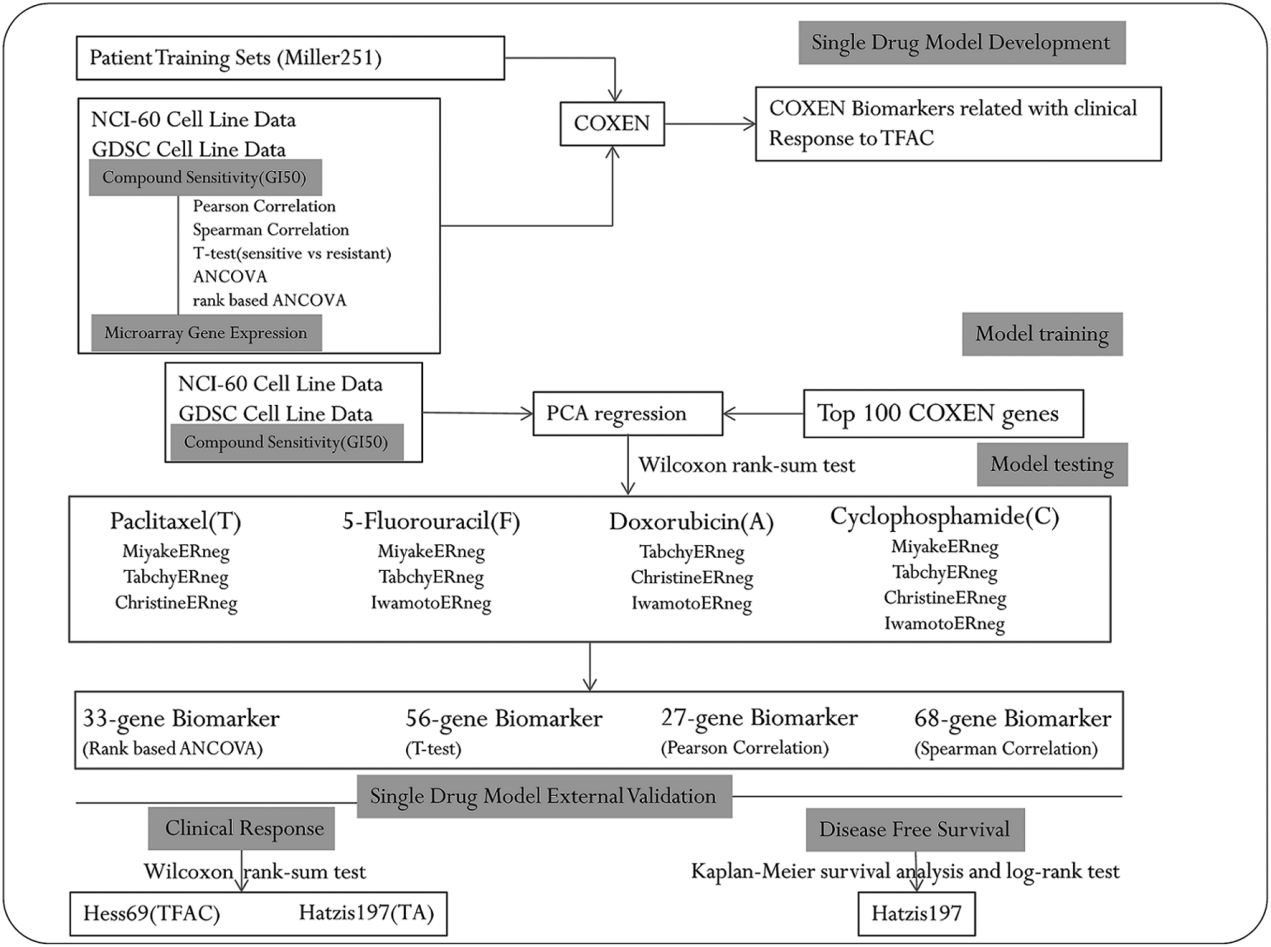
Schematic overview of biomarkers discovery and evaluation.

*In vitro* drug activity and microarray data for the 60 NCI cancer cell lines (NCI-60) were previously described elsewhere (Lee *et al*. 2007). In brief, drug sensitivity data for 50% growth inhibition (GI50) for the NCI-60 were obtained from the NCI Developmental Therapeutics Program (http://dtp.nci.nih.gov). NCI-60 expression profiling data with HG-U133A GeneChip arrays (Affymetrix) were obtained from the National Cancer Institute (http://discover.nci.nih.gov). Drug sensitivity data and 652 expression profiling data for GI50 for the GDSC-652 were also obtained from the Genomics of Drug Sensitivity in Cancer (http://www.cancerrxgene.org/). We also obtained and used seven different breast cancer cohorts for model development, selection and independent tests for our drug biomarker models (Table 1). The first cohort of 251 patients, Miller251 (Miller *et al*. 2005), was from a gene expression study on a general breast cancer population, which we used to select our drug biomarkers that were concordantly expressed between cancer cell lines and human breast cancer patients. This patient dataset was used only for concordant gene selection but not for any drug response evaluation and test. All patients in the other six breast cancer cohorts except Miller251 received neoadjuvant chemotherapy. We used the subsets of ER− patients in these patient datasets for our drug biomarker analysis and validation in this study. Response was categorized as a pathological complete response (pCR) or residual invasive disease (RD). Christine171 dataset consists of 279 (171 ER−) primary invasive breast cancer patients who received neoadjuvant doxorubicin/cyclophosphomide and ixabepilone or paclitaxel (Horak *et al*. 2013). Tabchy79 dataset includes the 178 patients (79 ER−) with clinical stages I–III, who were randomly assigned to receive either weekly paclitaxel ×12 followed by FAC (5-fluorouracil/doxorubicin/cyclophosphomide) ×4 or only FAC ×6 neoadjuvant chemotherapy (Tabchy *et al*. 2010). Iwamoto55 dataset includes 97 breast cancer patients (55 ER−) treated with four courses of FAC or FEC (5-fluorouracil/epirubicin/cyclophosphomide) chemotherapy (Iwamoto *et al*. 2011). Miyake44 dataset of 115 patients (44 ER−) was used to investigate whether GSTP1 expression was associated with resistance to neoadjuvant paclitaxel followed by 5-fluorouracil/epirubicin/cyclophosphomide (T-FEC) (Miyake *et al*. 2012). Hess69 is derived from Hess133 and Hess100, the 233 patients, (69 ER−) who received 24 weeks of sequential paclitaxel and fluorouracil-doxorubicin-cyclophosphomide (TFAC) preoperative chemotherapy at the M.D. Anderson Cancer Center (Hess *et al*. 2006, Lee *et al*. 2010). Patients in Hatzis197 cohort of 488 patients (197 ER−) received sequential taxane and anthracycline-based regimens and were followed to capture the durations of their recurrent-free survival (RFS) (Hatzis *et al*. 2011). This study was not required to obtain an approval from Moffitt Institutional Review Board and Ethics Committee as the study was based on deidentified retrospective patient data previously published at public domains. For the patient data used in this study, full consent has been obtained from each of the patients for the research use and purpose of their deidentified data in the previous studies (Miller *et al*. 2005, Hess *et al*. 2006, Tabchy *et al*. 2010, Hatzis *et al*. 2011, Iwamoto *et al*. 2011, Miyake *et al*. 2012, Horak *et al*. 2013). A schematic diagram is shown for the procedures of our biomarker model discovery, selection and validation based on these datasets in Fig. 1.

**Table 1:** Breast cancer patient cohorts used for model evaluation and indpendent test.

	COXEN	Biomarkers selection and evaluation				Independent model validation			
	Miller251	Christine171	Tabchy79	Iwamoto55	Miyake44	Hess69	Hatzis197	Hess48	Hatzis170
ER status	ER+/−	ER−	ER−	ER−	ER−	ER−	ER−	TN	TN
Platform	HG-U133A	HG-U133plus2	HG-U133A	HG-U133A	HG-U133plus2	HG-U133A	HG-U133A	HG-U133A	HG-U133A
*N*	251	171	79	55	44	69	197	48	170
Stage									
I	–	–	–	–	0	0	11	0	8
II	–	–	–	–	37	11	97	8	79
III	–	–	–	–	7	58	59	40	53
IV	–	–	–	–	0	0	30	0	30
Response									
RD	–	110	60	37	25	38	129	27	113
pCR	–	58	19	18	19	31	68	21	57
Others	–	3	–	–	–	–	–	–	–
Median age (range)	64 (28–93)	46 (25–73)	50 (29–73)	50.5 (30–75)	55.5 (28–70)	53 (32–75)	48 (24–75)	51.5 (31–75)	49 (24–75)
PR (+/−)	–	15/156	5/74	–	0/44	8/61	20/177	0/48	0/170
HER2 (+/−)	–	20/151	17/62	–	18/26	50/18	4/187	0/48	0/170
Drugs	–	AC + Taxol or AC + Ixabepilone	FAC,TFAC	FAC	TFC	TFAC	TA	TFAC	TA

### Drug sensitivity biomarker discovery

NCI-60 and GDSC-652 cell line datasets were used for the initial biomarker selection. We identified the initial gene biomarkers highly correlated with *in vitro* drug sensitivity or significantly differentially expressed between each drug’s sensitive and resistant cell lines for each of the four drugs. Several different statistical methods have been used for this analysis, including Pearson product–moment correlation, Spearman’s rank-order correlation, Welch’s two-sample *t*-test by grouping the cell line into sensitive and resistant with median GI50 as the cutoff, analysis of covariance (ANCOVA) and rank-based ANCOVA, whose results were compared to choose the biomarker discovery strategy ideal for each drug. We used and compared these multiple analysis approaches because molecular biomarkers could exhibit different association patterns with drug activities that could be quantitatively captured by different methods. The ANCOVA was leveraged to assess the degree of correlation between gene expression and drug activities while taking into account differences in overall drug activities among histological types of cell lines.

We next used the COXEN (COeXpression ExtrapolatioN) correlation analysis as reported elsewhere (Lee *et al*. 2007, Lee *et al*. 2010). COXEN summarizes the degree of concordance of expression regulation between two different cancer systems to effectively select genes that are consistently expressed and functionally involved between the two different cancer panels by evaulating each gene’s second-order correlation (or correlation of correlations) – each gene’s coexpression patterns with all the other genes and then between the two cancer sets (i.e., NCI-60 vs Miller251, GDSC-652 vs Miller251) (Williams *et al*. 2009, Smith *et al*. 2010). For this anlysis, we used a dataset of 251 patients, Miller251, which well represented a general breast cancer population; this set was used only for this purpose in our study. Triaging the initial *in vitro* chemosensitivity biomarkers into the biomarkers that were also relevant to human patients by COXEN, we identified the COXEN genes with the highest overall correlation of (consistent) expression patterns between the two different cancer panels – cell lines and human patient tumors.

### Chemotherapy response biomarker modeling and selection

The selected COXEN genes were then used to obtain individual drug biomarker models that could predict chemosensitivity and clinical response to each chemotherapy drug. A prediction score of chemosensitivity is a linear combination of expression values of multiple COXEN genes on cancer cell lines. Briefly, COXEN analysis resulted in a probe set of 27–68 genes per compound that provided the optimal biomarker prediction for each drug. We applied principal component regression analysis sequentially on the COXEN biomarker set, which was obtained from NCI-60 and GDSC-652 cell line training datasets with the GI50 as the dependent and COXEN biomarker genes as the independent variables. In this *in vitro*-based model training, we did not use any clinical information or expression data from the patient data sets, which were later used for the evaluation of the models, maintaining strict independence between training and evaluation data sets. The final biomarker model of each drug was selected from the evaluations on four ER− breast cancer patient datasets: Christine171, Tabchy79, Iwamoto55 and Miyake44, which were necessary to evaluate all four drug models. We obtained the optimal biomarker model for each drug by selecting the most significant biomarker model consistently on the majority of evaluation patient datasets. For a consistent and practical use of these statistical prediction values without loss of information, the predicted scores of individual drug models were converted into rank-based percentile scores between zero and one. A multivariate logistic regression analysis was then performed to generate the prediction model for differentiating pCR from RD for each relevant combination chemotherapy by combining the prediction scores from individual drug biomarker models under the assumption that the individual compounds in the combination acted independently. The prediction scores from the logistic regression model were used to evaluate the performance of the biomarker prediction on each combination therapy. All statistical analysis was performed with statistical software R (RStudio, version 1.0.143).

### Validation of individual drug and combined drug predictors

For each final drug biomarker model, we carried out an external validation to confirm its objective predictability for the chemotherapy response and DFS of ER− and TN breast cancer patients. For this validation test, the final predictors of the four drugs were applied to two independent breast cancer cohorts: Hess69 for the TFAC combination chemotherapy and Hatzis197 for the TA combination chemotherapy used to treat the patients in each study. Performance of these predictors was first evaluated by testing for a significant difference in the prediction scores between pathological complete response (pCR) vs residual disease (RD) patient groups using a non-parametric Wilcoxon rank-sum test. Kaplan–Meier survival analysis and log-rank test were next performed to understand the prediction performance for patient survival by combining these four or two drug predictors with multivariate logistic regression models.

## Results

### Identification of drug response biomarkers

As described earlier, NCI-60 and GDSC-652 cancer cell line panels were used for initial drug biomarker discovery and four patient datasets were used for drug model evaluation including Christine171, Tabchy79, Iwamoto55 and Miyake44. From these evaluations, our optimal biomarker models were obtained for paclitaxel, doxorubicin, cyclophosphamide and 5-fluorouracil. For paclitaxel, the biomarker model with 33 genes was selected by rank-based ANCOVA analysis. For 5-fluorouracil, the model with 56 genes was selected by *t*-test. For doxorubicin, the model with 27 genes was selected from GDSC-652 breast cancer cell lines by Pearson correlation analysis. As for the cyclophosphamide, the model with 68 genes was selected by Spearman correlation analysis.

### Independent drug biomarker test for chemotherapy response prediction

**Figure 2:**
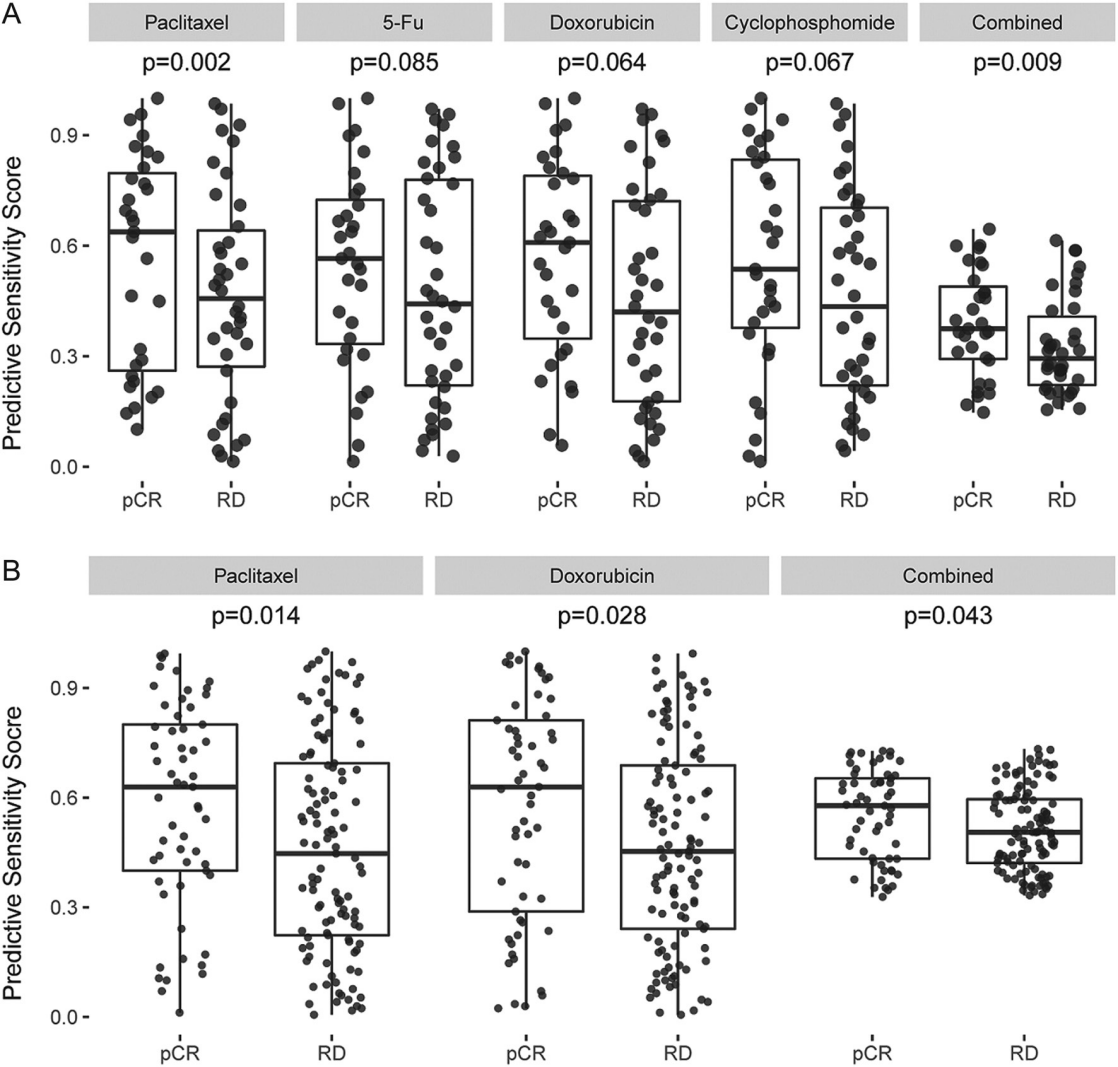
Chemotherapy response evaluation of single and combined drug models for the TN group in the Hess and Hatzis cohorts, respectively. (A) Paclitaxel, 5-fluorouracil, doxorubicin, cyclophosphamide and combined model evaluation for TN group in the Hess cohort, (B) paclitaxel, doxorubicin and combined model evaluation for TN group in the Hatzis cohort.

Hatzis197 and Hess69, which were not used for any of our discovery and model development, were chosen as independent datasets to validate the prediction performance of our drug biomarkers. In this analysis, we validated our biomarker models on the ER− and the TN subgroups of these cohorts, the latter group being a subset of the former group. The ER− groups consisted of 197 patients in the Hatzis cohort and 69 patients in the Hess cohort. Biomarker models for paclitaxel and doxorubicin drug models consistently significantly (or marginally significantly) predicted the pCR patients from the RD patients both for ER− and TN subgroups of Hatzis197 and for TN subgroup of Hess69 (Fig. 2 and Table 2). However, their prediction power became weaker for the ER− group in Hess69, likely due to its small sample size with more mixed outcomes of all ER− patients in this cohort. Nevertheless, all single drug biomarker models for paclitaxel, 5-fu, doxorubicin and cyclophosphamide significantly (or marginally significantly) predicted pCR patients from RD patients for the TN subgroup in both sets. Also, once these four (or two) drug predictors are combined, their combined drug models consistently showed significant prediction score for both ER− and TN groups of these cohorts. Therefore, the individual drug and combined biomarker models well predicted chemotherapy responses of the two patient cohorts who were treated with different combination chemotherapies.

**Table 2:** Biological functions of COXEN biomarker genes relevant to mechanisms of individual drugs.

Drugs	Function	Genes
Paclitaxel	Cell cycle, cell division, cell proliferation and differentiation	*CCND1, NUP160, DDRGK1, ELK4*
	Transcription regulation	*ELK4, NOC2L, WWC1, CCND1, MAP3K9, ZBTB25*
Doxorubicin	DNA-templated, regulation of transcription	*GLI3, H2AFX, CHTOP, PPP3CA, ZNF764*
	Transition of mitotic cell cycle	*PPP3CA, PPM1D, ID4*
Cyclophosphamide	Phosphatase activity	*TSKS, PPP1R16B*
	Immune response	*ICAM3, PSMB10, PTK2B, IL2RG*
5-Fluorouracil	DNA replication	*CDT1, RFC5, TIMELESS, TWNK, PRIM1, DNAJA3*
	DNA damage response	*MRPS35, CD44, RUVBL1, CHEK2, PEA15*

### Drug biomarker prediction for survival time

**Figure 3:**
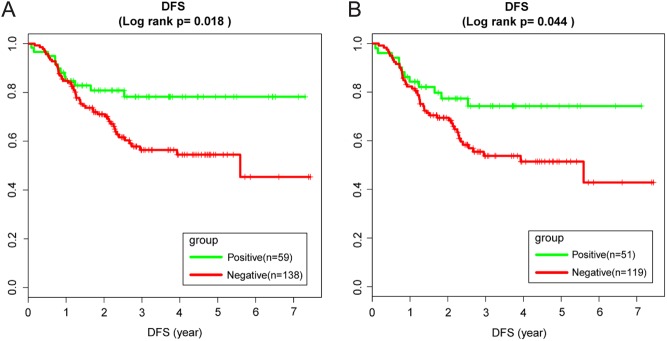
Disease-free survival time (DFS) for the ER− and TN groups of patients in the Hatzis cohort: (A) ER− group, (B) TN group.

We also examined if these drug biomarker models provided a significant difference in disease-free survival time (DFS) for the ER− and TN groups of patients in the Hatzis cohort (Fig. 3). Similar patterns were found, but survival analysis was unreliable for the Hess cohort due to its small sample size of ER− patients. The patients were treated with combination chemotherapy of paclitaxel and doxorubicin in the Hatzis cohort, so this combined drug model was used for the survival analysis. In order to evaluate a survival time difference between predicted responders, or ‘COXEN positive group,’ and predicted non-responders, or ‘COXEN negative group’, we performed the Kaplan–Meier survival analysis for the ER− and TN groups in Hatzis197. Similar to the original study on this cohort, we used the cutoff value of our drug model prediction with top 30% as the COXEN-positive patients and the rest as the COXEN-negative patients in this analysis. We found that DFS was significantly longer for the COXEN-positive group than the COXEN negative group with median DFS >84 month vs 67.08 month (log-rank test *P* = 0.018). A consistent DFS difference was also found for the TN group (log-rank test *P* = 0.044).

## Discussion

ER receptor status has been widely recognized as an important prognosis factor in breast cancer. In particular, ER− breast cancer patients are often more sensitive to chemotherapy, but generally show earlier recurrence and unfavorable prognosis and survival compared to ER+ breast cancer patients. However, as these ER− patients have shown highly heterogeneous responses to different chemotherapies, it has been challenging to select most beneficial chemotherapy treatments for individual patients with ER− breast cancer. Previous studies have shown potential of genomic biomarkers for predicting outcomes of these breast cancer patients treated with chemotherapy, but their prediction capabilities were restricted to each specific combination chemotherapy, which was exactly used to treat the patients in each study. In this study, we have simultaneously developed biomarker signatures for four chemotherapy agents based on a common platform of genomic expression profiling: paclitaxel (T), 5-fluorouracil (F), doxorubicin (A) and cyclophosphamide (C) to predict responses and survival of ER− breast cancer patients treated with combination chemotherapies. Flexibly combining these individual drug signatures, we predicted response and survival of two independent cohorts of patients who were treated with different drug combinations of neoadjuvant chemotherapy. From these independent tests, we were able to validate significant prediction performance of both single and combined drug biomarker models for ER− and TN breast cancer patients in these cohorts.

Many of these biomarkers were found to have known functions relevant to each drug’s mechanism of action (Table 3). For instance, COXEN biomarkers of paclitaxel include *CCND1* (Baldin *et al*. 1993), *NUP160* (Chakraborty *et al*. 2008), *DDRGK1* (Liu *et al*. 2017) and *ELK4* (Peng *et al*. 2016), which are well-known regulators of cell cycle and cell division. Regulation of these genes can thus block the progression of mitosis and can activate the mitotic checkpoint, which triggers apoptosis or reversion to the G-phase of the cell cycle. For doxorubicin, *GLI3* (Lauth *et al*. 2007), *H2AFX* (Podhorecka *et al*. 2010) and *ZNF764* (Latorre *et al*. 2012) are involved in the transcription regulation of topoisomerase II, an enzyme which relaxes supercoils in DNA for transcription. As for the biomarkers in cyclophosphamide model, *TSKS* (Silva *et al*. 2014) and *PPP1R16B* (Boratko *et al*. 2015) are related with phosphatase activity, while *ICAM3* (Green *et al*. 2014), *PSMB10* (Morales Poole *et al*. 2017), *PTK2B* (Kremer *et al*. 2014) and *IL2RG* (Breen *et al*. 2016) are related with immune response. 5-Fu interrupts the action of thymidylate synthase, thereby blocking synthesis of the pyrimidine thymidine, a nucleoside required for DNA replication; this drug’s COXEN biomarkers such as *CDT1* (Shibata *et al*. 2014), *RFC5* (Pearl *et al*. 2015) and *TIMELESS* (Young *et al*. 2015) were known to be directly related to this mechanism. All the biomarkers which could predict the response to TFAC were listed in additional file1.

**Table 3:** Performance of single and combined drug models to predict pathologic response for ER− and triple-negative breast cancer patients.

Subtype (*n*)	Paclitaxel		5-Fluorouracil		Doxorubicin		Cyclophosphamide		Combined	
	AUC	*P*-value	AUC	*P*-value	AUC	*P*-value	AUC	*P*-value	AUC	*P*-value
Hatzis										
ER− (197)	0.639	0.001			0.606	0.015			0.637	0.002
TN (170)	0.615	0.014			0.603	0.028			0.595	0.043
Hess										
ER− (69)	0.596	0.176	0.565	0.356	0.612	0.113	0.599	0.161	0.635	0.056
TN (48)	0.755	0.002	0.647	0.085	0.658	0.064	0.656	0.067	0.72	0.009

Currently, predictive biomarker models for cancer therapeutics often generate false negative results in over 10% of responsive patients (or negative predictive value (NPV) lower than 90%); for example, >10% of ER− patients have been found to be sensitive to endocrine therapy but not recommended to be treated with it based on their ER type (Manna & Holz 2016). Individual patient tumors can be responsive to multiple treatment options. If a biomarker model is separately used only for a single therapy, this risk probability of false negative prediction cannot easily be improved for overall cancer treatment. However, if we simultaneously use biomarker models for two (or more) alternative treatment options, then the risk probability of falsely predicting negatively for both treatment options when they both are effective (so the patient is falsely excluded for both effective treatments) will simply decrease. We have summarized the risk probability of falsely predicting negatively by our individual drug biomarker models, or 1-NPV, for the ER and TN groups of the Hess and Hatzis cohorts (Supplementary Table 1, see section on supplementary data given at the end of this article). To show its mathematical effects, hypothetically assuming that they are used alternatively and that their drug effects are largely independent, the risk probability of falsely predicting negatively for two effective drugs can then be smaller than 9% if a patient is responsive to two drug options. The risk probability for excluding a patient from all effective drugs can be lower than 2% if the patient is responsive to three drug options, which will continuously decrease as we use more alternative treatment biomarkers together. The ultimate goal of these biomarkers is to provide a rational basis in prioritizing a particular agent from multiple available chemotherapeutic agents which are used largely in a trial and error manner.

There are several limitations of our current study. First, similar to the previous studies, we used the top 30% prediction score as the cutoff for dividing predicted responder vs predicted non-responder patients for our survival analysis. We chose such a fixed cutoff to avoid a multiple comparisons pitfall when the best cutoff value is sought from many possible cutoff values. However, we believe that there would be a better cutoff value, which will require other independent patient sets to derive and validate such an optimal cutoff value. Another limitation of our study is the lack of data from patients with single agent therapies, which inhibited us from examining single drug prediction performance more accurately. Our current combined drug predictors also ignore multi-drug interaction effects on the patients from complex combination chemotherapies. We did not consider such drug interaction in the current study, because there were too many interaction terms among four drugs, which might result in model overfitting and saturation. We would not have sufficient power and data information to detect such drug interaction effects, either. We also found that there was a certain degree of cross-drug prediction power of our individual drug biomarker models as these chemotherapy drugs attack similar tumor cell activities such as rapid cell division. However, we confirmed that their prediction power for other drugs was drastically lower than that for the specific drug of each model on the NCI-60 cell line panel. Thus, our results showed that these individual drug biomarkers were more specifically predictive of target drug responses. We tested if our biomarker models were continuously predictive of DFS time using a Cox regression analysis, which was not found to be significant. It can be due to a violation of the Cox regression model assumption from our drug biomarker models and prediction score distributions. Another possibility is that the benefit of our predicted sensitive drugs might have been obtained for a certain top proportion of sensitive patients rather than for all patients based on a proportional hazard ratio continuously over time.

It is also somewhat difficult to discern if our biomarker models can predict response to chemotherapies beyond favorable patient survival in this study. This is due to the fact that all patients were treated with the same combination chemotherapies in each study of the patient data sets in our current study. We, however, partially dealt with a similar question for alternative combination chemotherapies in the previous ovarian cancer study in which we used a large TCGA set of ~450 ovarian cancer patients who were treated with diverse different drug combinations (Kim *et al*. 2014). In this study, we showed a significant survival difference between the matched patients who were treated with drug combinations with positive biomarker prediction and the unmatched patients who were not treated with drug combinations with positive biomarker prediction. We further confirmed these matched and unmatched groups of patients were identical for other clinical prognostic factors other than their treatment selections in the study. Therefore, the survival difference of the two groups could be cautiously inferred due to the treatment selection independently from their prognostic factors. We could not use such a large breast cancer patient cohort where patients were treated with diverse chemotherapies to examine this kind of survival difference in the current study; presently, TCGA breast cancer set has insufficient clinical annotation for their treatment and outcome information.

Development and clinical translation of molecular biomarker models has been challenging for a number of reasons. The biggest difficulty is that it is quite lengthy and extremely costly to confirm such biomarker models in clinical settings. Moreover, as seen in controversial scientific reports a few years ago, there is a significant concern in cancer science community about a great danger of false discovery and model overfitting of such biomarker models. Also, clinical use and regulation on such biomarker assays have not yet been clearly defined. Despite these challenges the development and clinical use of these biomarker models are extremely important for improving overall outcome of cancer patients in the era of precision medicine with an exploding number of novel therapies. We believe our single drug biomarker-based development has a potential to greatly improve efficiency of developing and translating them into clinical practice since we only need to validate and translate biomarker models for individual drugs rather than biomarker models for their numerous combination therapies. This capability may allow us to even select new drug combinations most beneficial to individual patients with ER− breast cancer. These questions will need to be investigated in the future. The validation carried out in this study was retrospective validation of the statistical models that had been produced. Although our aim in this study is to produce predictive models of clinical significance, it is important to note that the prospective use of our models in a clinical context has not been developed in detail and would require independent, prospective, clinical validation.

## Supplementary Material

Supporting Table 1

## Declaration of interest

The authors declare that there is no conflict of interest that could be perceived as prejudicing the impartiality of the research reported.

## Funding

This work was supported in part by the NSFC/China (81402175) funding and the Moffitt Cancer Center.
