# Supplementary material for: Single drug biomarker prediction for ER− breast cancer outcome from chemotherapy
Source: Endocr Relat Cancer. 2018 Mar 29;25(6):595–605. doi: 10.1530/ERC-17-0495 (PMC5920016; doi:10.1530/ERC-17-0495)
Supplement: Supporting Table 1 [file erc-25-595-t001.pdf]

Supplementary Materials

	Paclitaxel	5-Fu	doxorubicin	Cyclophosphamide
Dataset (n)	1- NPV	1-NPV	1-NPV	1-NPV
Hess-ER- (197)	33.3%	33.3%	35.0%	25.0%
Hess-TN (170)	17.4%	27.3%	9.1%	33.4%
Hatzis-ER- (69)	16.9%		26.8%	
Hatzis-TN (48)	18.3%		25.7%	

Supplementary Table 1. Risk Probability for Falsely Excluding Responsive Patients based on Single Drug Biomarker Models. The risk probabilities for falsely excluding responsive patients are summarized for each of our single drug biomarker models. Individual patient tumors can be responsive to multiple treatment options. To show its mathematical effects, hypothetically assuming that these drug effects are largely independent and that they could be alternatively used, e.g, for recurrent MBC patients, the risk probability of falsely predicting negatively for two effective drugs can be smaller than 9% (=0.273*0.334) from the two largest (1-NPV) drug models (5-Fu and Cyclophosphamide) in the Hess TN subgroup if a patient is responsive to two drugs, and smaller than 2% if a patient is responsive to three drug options. This risk probability will continuously decrease as we use more alternative treatment biomarker models together.
